# The effect of non-optimal lipids on the progression of coronary artery calcification in statin-naïve young adults: results from KOICA registry

**DOI:** 10.3389/fcvm.2023.1173289

**Published:** 2023-07-17

**Authors:** Heesun Lee, Hyo-Jeong Ahn, Hyo Eun Park, Donghee Han, Hyuk-Jae Chang, Eun Ju Chun, Hae-Won Han, Jidong Sung, Hae Ok Jung, Su-Yeon Choi

**Affiliations:** ^1^ Department of Internal Medicine, Seoul National University College of Medicine, Seoul, Republic of Korea; ^2^ Division of Cardiology, Healthcare System Gangnam Center, Seoul National University Hospital, Seoul, Republic of Korea; ^3^ Division of Cardiology, Cardiovascular Center, Seoul National University Hospital, Seoul, Republic of Korea; ^4^ Department of Imaging, Cedars-Sinai Medical Center, Los Angeles, CA, United States; ^5^ Division of Cardiology, Yonsei Cardiovascular Center, Yonsei University Health System, Seoul, Republic of Korea; ^6^ Division of Radiology, Seoul National University Bundang Hospital, Seongnam, Republic of Korea; ^7^ Department of Internal Medicine, Gangnam Heartscan Clinic, Seoul, Republic of Korea; ^8^ Division of Cardiology, Heart Stroke and Vascular Institute, Samsung Medical Center, Seoul, Republic of Korea; ^9^ Division of Cardiology, Department of Internal Medicine, College of Medicine, Seoul St. Mary’s Hospital, The Catholic University of Korea, Seoul, Republic of Korea

**Keywords:** dyslipidemia, atherosclerosis, coronary calcification, prevention, young adults

## Abstract

**Background:**

Despite the importance of attaining optimal lipid levels from a young age to secure long-term cardiovascular health, the detailed impact of non-optimal lipid levels in young adults on coronary artery calcification (CAC) is not fully explored. We sought to investigate the risk of CAC progression as per lipid profiles and to demonstrate lipid optimality in young adults.

**Methods:**

From the KOrea Initiative on Coronary Artery calcification (KOICA) registry that was established in six large volume healthcare centers in Korea, 2,940 statin-naïve participants aged 20–45 years who underwent serial coronary calcium scans for routine health check-ups between 2002 and 2017 were included. The study outcome was CAC progression, which was assessed by the square root method. The risk of CAC progression was analyzed according to the lipid optimality and each lipid parameter.

**Results:**

In this retrospective cohort (mean age, 41.3 years; men 82.4%), 477 participants (16.2%) had an optimal lipid profile, defined as triglycerides <150 mg/dl, LDL cholesterol <100 mg/dl, and HDL cholesterol >60 mg/dl. During follow-up (median, 39.7 months), CAC progression was observed in 434 participants (14.8%), and more frequent in the non-optimal lipid group (16.5% vs. 5.7%; *p *< 0.001). Non-optimal lipids independently increased the risk of CAC progression [adjusted hazard ratio (aHR), 1.97; *p *= 0.025], in a dose-dependent manner. Even in relatively low-risk participants with an initial calcium score of zero (aHR, 2.13; *p *= 0.014), in their 20 s or 30 s (aHR 2.15; *p *= 0.041), and without other risk factors (aHR 1.45; *p *= 0.038), similar results were demonstrable. High triglycerides had the greatest impact on CAC progression in this young adult population.

**Conclusion:**

Non-optimal lipid levels were significantly associated with the risk of CAC progression in young adults, even at low-risk. Screening and intervention for non-optimal lipid levels, particularly triglycerides, from an early age might be of clinical value.

## 1. Introduction

Dyslipidemia is one of the major cardiovascular risk factors, and attainment of the optimal lipid levels can prevent atherosclerotic cardiovascular disease (ASCVD) and is associated with a better prognosis (1–4). Meanwhile, dyslipidemia and related ASCVD in young adults have often been overlooked by both physicians and patients due to the perception that blood lipid levels tend to increase with age and ASCVD generally occurs in middle-aged and elderly individuals. Recent literature, however, has stated that the cumulative exposure of lipids in an individual’s lifetime is contributory to the initiation and progression of ASCVD, and thus, early screening for dyslipidemia and maintenance of optimal lipid levels from a younger age are recommended to preserve cardiovascular health (1, 5–8). The prevalence of dyslipidemia in young adults varies across regions but is increasing faster than expected. For instance, approximately 36% of US adults aged 20–29 years and 43% of those aged 30–39 years met abnormal lipid levels defined by *the National Cholesterol Education* Program (9). Similarly, as per the report by *the Korean Society of Lipid and Atherosclerosis*, 19% of adults in their 20 s and 28% in their 30 s suffered from dyslipidemia (10). Non-optimal lipid levels in young adults are known to increase the risk of ASCVD in the short-term and in the long-term (11–14).

To explore the relationship between dyslipidemia during young adulthood and ASCVD, several studies have utilized coronary artery calcium score (CACS) in conjunction with the lipid profile (15, 16). CACS reflects the burden and severity of atherosclerosis in coronary arteries, with relatively low radiation exposure and cost (17). Moreover, the progression of coronary artery calcification (CAC), as assessed using repeated measurements of CACS, is a strong predictor of further ASCVD (18). However, prior studies have mainly focused on advanced coronary calcification later in life based on non-optimal lipid levels during young adulthood (16, 19). Considering the recently reinforced importance of early management of dyslipidemia and the role of CACS (6, 7), we aimed to investigate the risk of CAC progression according to the lipid profile in young adults.

## 2. Materials and methods

### 2.1. Study population and design

**Figure 1:**
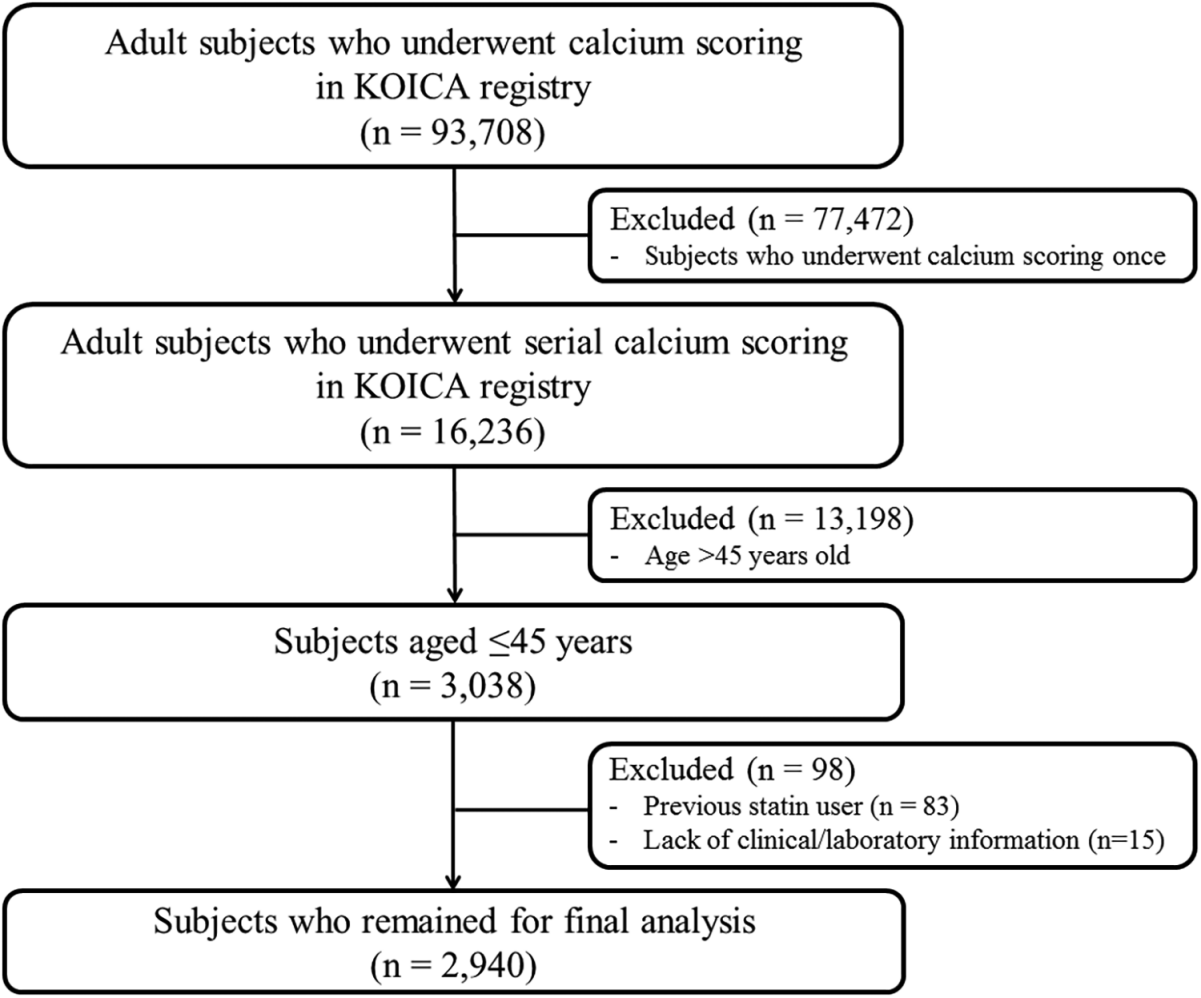
Schematic flowchart of the study population. KOICA, Korea Initiative on Coronary Artery calcification.

Data from the KOrea Initiative on Coronary Artery calcification (KOICA) registry were used in this study. The KOICA registry is an observational, retrospective, single ethnicity, multi-center registry of self-referred adults who underwent coronary artery calcium scan as a part of routine health check-ups at six healthcare centers in Korea, as described previously (20, 21). Between December 2002 and March 2017, a total of 93,708 individuals were consecutively enrolled. From the initial cohort, those who underwent at least two calcium scans were recruited (*n* = 16,236). Among them, those aged ≤45 years at the initial examination (*n* = 3,038) were selected. After excluding those with prior use of statins (*n* = 83) and those without available clinical or laboratory information (*n* = 15), we finally analyzed 2,940 statin-naïve young adults in this study (Figure 1). The study protocol conformed to the guidelines of the 1975 Declaration of Helsinki, as reflected by an *a priori* approval by the Institutional Review Boards of each participating center (H-1406-053-587). The requirement for written informed consent was waived due to the retrospective nature of the study.

### 2.2. Clinical and laboratory evaluation

Information on sociodemographic profiles and risk factors were acquired at the time of each patient visit to the healthcare centers. Anthropometric information, including height, weight, waist circumference, and blood pressure, was collected by a trained nurse. A self-reported questionnaire was used to assess smoking, alcohol status, family history of premature cardiovascular disease, and previous medical history including hypertension, diabetes mellitus and medication use. Laboratory evaluations were performed at the Department of Laboratory Medicine of each center, and included serum total cholesterol (TC), triglycerides, high-density lipoprotein cholesterol (HDL-C), low-density lipoprotein cholesterol (LDL-C), fasting glucose, glycated hemoglobin, serum creatinine, high-sensitivity C-reactive protein (hs-CRP), and *γ*-glutamyl transpeptidase after a minimum of 8 h fasting. The optimal lipid levels were defined when all of the following criteria were satisfied: triglycerides <150 mg/dl, LDL-C < 100 mg/dl, and HDL-C > 60 mg/dl (16, 22, 23). The non-optimal lipid levels were defined when any of optimal lipid criteria was not satisfied. Furthermore, the non-optimal lipid levels were divided into two categories: (1) 150 ≤ triglycerides <200 mg/dl, 100≤ LDL-C < 130 mg/dl, or 40< HDL-C ≤ 60 mg/dl as suboptimal level, (2) triglycerides ≥200 mg/dl, LDL-C ≥ 130 mg/dl, or HDL-C ≤ 40 mg/dl as abnormal level.

### 2.3. Measurement of CAC and its progression

All subjects underwent non-contrast calcium scans using a >16-detector row CT scanner (SOMATOM Sensation 16, Siemens, Forchheim, Germany; Brilliance 40, Philips Medical Systems Inc., Amsterdam, the Netherlands; LightSpeed VCT 64, GE, Boston, USA; Brilliance iCT 256, Philips Medical Systems Inc., Amsterdam, the Netherlands). A standard protocol was applied, with a prospective or retrospective electrocardiography-triggering and image acquisition initiated at 70% of the cardiac cycle for motion-free images of coronary arteries (24). Pixels exceeding 130 Hounsfield units were identified as a discrete calcified focus, and CACS was automatically calculated by the summation of all calcification scores using the Agatston scoring system (in units) based on the area density of each calcified focus. The CACS was graded as follows: 0, 1–10, 11–99, and ≥100.

The primary endpoint of the study was the progression of CAC during follow-up, defined as a difference of ≥2.5 between the square roots (√) of the baseline and follow-up CACS (Δ√transformed CAC) to minimize the effect of inter-scan variability (25).

### 2.4. Statistical analysis

Continuous variables were presented as mean ± standard deviation (SD) or median and inter-quartile range, while categorical variables were expressed as numbers and percentages. Intergroup differences of continuous variables were compared using Student’s *t-test* for independent samples or the Mann–Whitney test, and categorical variables were compared using the *χ*^2^ test or Fisher’s exact test. Conventional risk factors and CACS were compared according to lipid abnormalities. To identify predictors responsible for CAC progression and evaluate the risk of CAC progression for each lipid variable, univariable and multivariable Cox proportional hazard models were employed. The risk of CAC progression was expressed as a hazard ratio (HR) and the corresponding 95% confidence interval (CI). The chronological trend for the probability of CAC progression according to the lipid profiles was obtained by Kaplan-Meier analysis with the log-rank test. In addition, the following subgroup analyses were conducted to examine the risk of CAC progression according to lipid abnormalities: (1) in participants in their 20 s and 30 s, (2) in those with an initial CACS of zero, and (3) in those without any other conventional risk factors, except dyslipidemia. All analyses were performed using SPSS version 22.0 (IBM Corp., Armonk, NY, USA). A value of two-sided *p* < 0.05 was considered statistically significant.

## 3. Results

### 3.1. Baseline characteristics of the study population

In the present cohort consisting of 2,940 young adults aged ≤45 years from the KOICA registry (mean age, 41.3 years; men 82.4%), only 477 participants (16.2%) had an optimal lipid profile at baseline. Among the rest with non-optimal lipids, one-third (*n* = 884, 35.9%) had suboptimal lipid levels, and the remaining two-thirds (*n* = 1,579, 64.1%) showed abnormal lipid levels. The non-optimal lipid group was slightly older and more likely to be men than optimal lipid group. Conventional cardiovascular risk factors appeared to be more prevalent in the non-optimal lipid group than in their counterparts, while there was no significant difference in the self-reported family history of premature ASCVD between the two groups. The mean/median (triglycerides) values of the lipid profile were 204.2 (TC), 49.4 (HDL-C), 127.9 (LDL-C), and 134.0 (triglycerides) mg/dl in the non-optimal lipid group. The detailed baseline characteristics of the study participants are summarized in Table 1.

**Table 1:** Baseline clinical and imaging characteristics according to lipid levels during young adulthood.

Variables	Total (*n* = 2,940)	Optimal lipid levels (*n* = 477)	Non-optimal lipid levels (*n* = 2,463)	*p*
Clinical parameters				
Age, years	41.3 ± 3.7	40.7 ± 4.2	41.5 ± 3.6	0.003
Male	2,422 (82.4)	252 (52.8)	2,170 (88.1)	<0.001
BMI, kg/m^2^	24.5 ± 3.2	22.4 ± 2.8	24.9 ± 3.1	0.034
BMI ≥25 kg/m^2^	1,206 (41.0)	78 (16.4)	1,128 (46.1)	<0.001
WC, cm	85.2 ± 9.4	77.9 ± 9.4	86.3 ± 8.9	0.019
WC ≥90 cm (M) or 85 cm (F)	663 (22.6)	33 (6.9)	630 (25.6)	<0.001
Ever smoking	1,798 (61.2)	224 (48.6)	1,574 (66.9)	<0.001
Current smoking	779 (26.5)	98 (20.5)	681 (27.6)	<0.001
Moderate to heavy drinking	1,375 (46.8)	205 (43.0)	1,170 (47.5)	<0.001
Family history of premature CVD	500 (17.0)	71 (14.9)	429 (17.4)	0.178
Hypertension	425 (14.5)	41 (8.7)	384 (15.9)	<0.001
Diabetes mellitus	139 (4.7)	13 (2.7)	126 (5.1)	0.023
Prior use of antiplatelet agent	94 (3.2)	18 (3.8)	76 (3.1)	0.425
Statin after 1st calcium scan	199 (6.8)	2 (0.4)	197 (8.0)	<0.001
Systolic blood pressure, mmHg	118.4 ± 14.1	113.3 ± 13.5	119.4 ± 14.0	0.504
Diastolic blood pressure, mmHg	74.4 ± 10.9	70.2 ± 10.6	75.2 ± 10.8	0.354
Laboratory parameters				
Hb, g/dl	15.0 ± 1.4	14.0 ± 1.6	15.2 ± 1.3	<0.001
Total cholesterol, mg/dl	197.7 ± 33.9	163.9 ± 18.5	204.2 ± 32.3	<0.001
HDL-C, mg/dl	51.5 ± 12.8	62.4 ± 13.5	49.4 ± 11.5	0.001
Triglycerides, mg/dl	119.5 (80.0–177.0)	71.0 (56.5–95.0)	134.0 (93.0–193.0)	<0.001
LDL-C, mg/dl	120.7 ± 30.7	83.8 ± 11.9	127.9 ± 27.9	<0.001
Fasting glucose, mg/dl	94.6 ± 18.3	90.1 ± 12.3	95.5 ± 19.2	<0.001
HbA1c, %	5.6 ± 0.7	5.4 ± 0.4	5.6 ± 0.7	0.004
*γ*-GT, IU/L	44.2 ± 51.9	26.4 ± 29.5	47.6 ± 54.6	<0.001
Creatinine, mg/dl	0.9 ± 0.2	0.9 ± 0.2	0.9 ± 0.2	0.999
hs-CRP reactive protein, mg/L	0.6 ± 2.2	0.5 ± 1.9	0.6 ± 2.3	0.895
Imaging parameters—Baseline				
CACS	8.9 ± 47.1	5.3 ± 49.1	9.6 ± 46.7	0.004
Log (CACS + 1)	0.21 ± 0.54	0.07 ± 0.35	0.23 ± 0.56	<0.001
Categorical CACS				
0	2,449 (83.3)	449 (94.1)	2,000 (81.2)	<0.001
1–10	208 (7.1)	11 (2.3)	197 (8.0)	
11–100	213 (7.2)	12 (2.5)	201 (8.2)	
>100	70 (2.4)	5 (1.0)	65 (2.6)	
Imaging parameters—Follow-up				
CACS	29.2 ± 119.8	15.5 ± 128.5	31.9 ± 117.8	<0.001
Log (CACS + 1)	0.39 ± 0.77	0.13 ± 0.48	0.42 ± 0.79	<0.001
annualized ΔCACS	5.6 ± 26.0	3.4 ± 31.1	6.0 ± 24.9	0.045
Categorical CACS				
0	2,228 (75.8)	430 (90.1)	1,798 (73.0)	<0.001
1–10	174 (5.9)	11 (2.3)	163 (6.6)	
11–100	317 (10.8)	21 (4.4)	296 (12.0)	
>100	221 (7.5)	15 (3.1)	206 (8.4)	
Inter-scan period, months	39.7 (22.1–57.5)	40.2 (22.5–59.1)	39.6 (22.0–57.0)	0.675
CAC progression	434 (14.8)	27 (5.7)	407 (16.5)	<0.001

Values are mean ± standard deviation, median (interquartile range) or *n* (%).

BMI, body mass index; CAC, coronary artery calcification; CACS, coronary artery calcium scores; CCTA, coronary computed tomography angiography; CVD, cardiovascular disease; F, female; γ-GT, gamma-glutamyl transpeptidase; Hb, hemoglobin; HbA1c, glycated hemoglobin; HDL-C, high-density lipoprotein cholesterol; hs-CRP, high sensitivity-C reactive protein; LDL-C, low-density lipoprotein cholesterol; M, male; WC, waist circumference.

The mean CACS at baseline was 8.9, with approximately 83% of all participants having a CACS of zero, suggesting a low-risk population. Participants with non-optimal lipids tended to have a significantly higher CACS on average (5.3 in the optimal lipid group vs. 9.6 in the non-optimal lipid group, *p *= 0.004) and fewer cases with a CACS of zero (94.1% vs. 81.2%, *p *< 0.001), than those with optimal lipids (Table 1, Supplementary Figure S1).

### 3.2. CAC progression and its predictors

**Figure 2:**
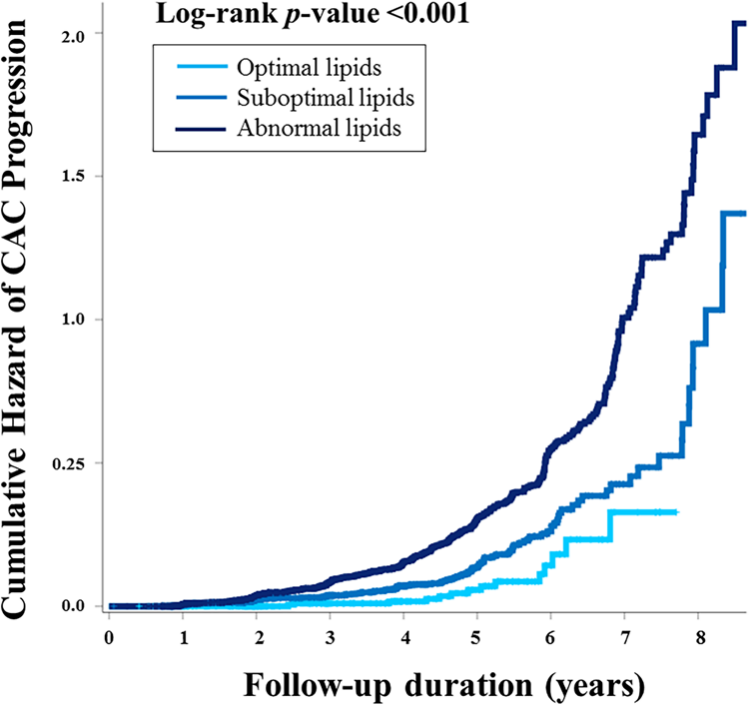
The cumulative risk of CAC progression according to lipid abnormality. Kaplan-Meier curves demonstrated the stratified risk increase according to lipid abnormality. Participants with optimal lipids had the best prognosis, suggesting “the more optimal for lipid during young adults, the better for preventing CAC progression.” CAC, coronary artery calcification.

The median interval between baseline and follow-up calcium scans in the present study was 39.7 months (interquartile range, 22.1–57.5), with no significant difference observed according to lipid profile (40.2 vs. 39.6 months, *p *= 0.675). At the follow-up, the mean CACS was 29.2 and three quarters of the participants still had a CACS of zero. When compared according to lipid profile, a significant difference in the absolute annualized changes of CACS was found between groups (3.4 vs. 6.0, *p *= 0.045). During the follow-up, CAC progression was observed in 434 participants (14.8%) and more frequent in the non-optimal lipid group [407/2,463 (16.5%)] than in the optimal lipid group [27/477 (5.7%)] (*p *< 0.001) (Table 2). Kaplan-Meier curves also demonstrated a gradual increase in the risk of CAC progression depending on lipid abnormality, with the worst prognosis in the abnormal lipid group (log-rank *p *< 0.001) (Figure 2).

**Table 2:** Predictors of CAC progression.

Variables	Univariable analysis		Multivariable analysis	
	Unadjusted HR (95% CI)	*p*	Adjusted HR (95% CI)	*p*
Non-optimal lipid level	2.62 (1.79–3.85)	<0.001	1.97 (1.18–3.34)	0.025
Suboptimal lipid level	1.59 (1.12–2.47)	0.013	1.21 (1.00–1.92)	0.049
Abnormal lipid level	3.02 (2.05–4.44)	<0.001	2.17 (1.41–3.51)	<0.001
Age (per 5 years increment)	1.45 (1.24–1.68)	<0.001	1.14 (0.97–1.34)	0.115
Male	6.56 (3.25–13.20)	<0.001	2.53 (1.21–5.29)	0.014
Obesity^a^	1.56 (1.29–1.88)	<0.001	1.17 (0.93–1.46)	0.180
Abdominal obesity^b^	1.87 (1.53–2.27)	<0.001	1.34 (1.06–1.69)	0.016
Ever smoking	1.30 (1.03–1.64)	0.025	1.03 (0.81–1.31)	0.794
Moderate to heavy drinking	1.58 (1.02–2.43)	0.041	0.96 (0.75–1.22)	0.732
Family history of premature CVD	1.16 (0.93–1.46)	0.191		
Hypertension	2.37 (1.93–2.90)	<0.001	1.48 (1.19–1.85)	<0.001
Diabetes mellitus	1.99 (1.49–2.66)	<0.001	1.46 (1.09–1.97)	0.012
Statin after 1st calcium scan	1.25 (0.97–1.61)	0.088		
Anemia	0.21 (0.05–1.84)	0.208		
hs-CRP ≥2.0 mg/L	1.55 (1.01–2.37)	0.046	1.99 (1.30–3.08)	0.002
Log (baseline CACS + 1)	2.95 (2.67–3.26)	<0.001	2.73 (2.43–3.07)	<0.001

^a^ Obesity was defined as BMI ≥25 kg/m^2^.

^b^ Abdominal obesity was defined as WC ≥90 cm (M) or 85 cm (F).

Multivariable analysis was adjusted for significant risk factors in the univariable analysis.

CI, confidence interval; HR, hazard ratio; other abbreviations are same as Table 1.

To evaluate significant predictors of CAC progression, Cox regression analyses were performed (Table 2). In the univariable analysis, age, male sex, obesity, abdominal obesity, smoking, alcohol drinking, hypertension, diabetes mellitus, increased hs-CRP, baseline CACS were significantly associated with the risk of CAC progression. Statin therapy after the initial calcium scan had a tendency to increase the risk of CAC progression but statistically insignificant (unadjusted HR 1.25, 95% CI 0.97–1.61, *p = *0.088). Non-optimal lipids at baseline demonstrated an increased risk of CAC progression by approximately 2.6-fold (unadjusted HR 2.62, 95% CI 1.79–3.85, *p *< 0.001). After adjusting for significant factors identified in the univariable analysis, conventional cardiovascular risk factors including age, male sex, abdominal obesity, hypertension, diabetes mellitus, increased hs-CRP, and log transformation of (baseline CACS + 1) remained predictors for CAC progression. Non-optimal lipids were independently associated with CAC progression (adjusted HR 1.97, 95% CI 1.18–3.34, *p *= 0.025) in young adults. Among participants with non-optimal lipid levels, there was a stepwise increase in the risk of CAC progression based on the degree of deviation from optimal lipid levels: adjusted HR 1.21 for the suboptimal lipid group vs. adjusted HR 2.17 for the abnormal lipid group.

**Figure 3:**
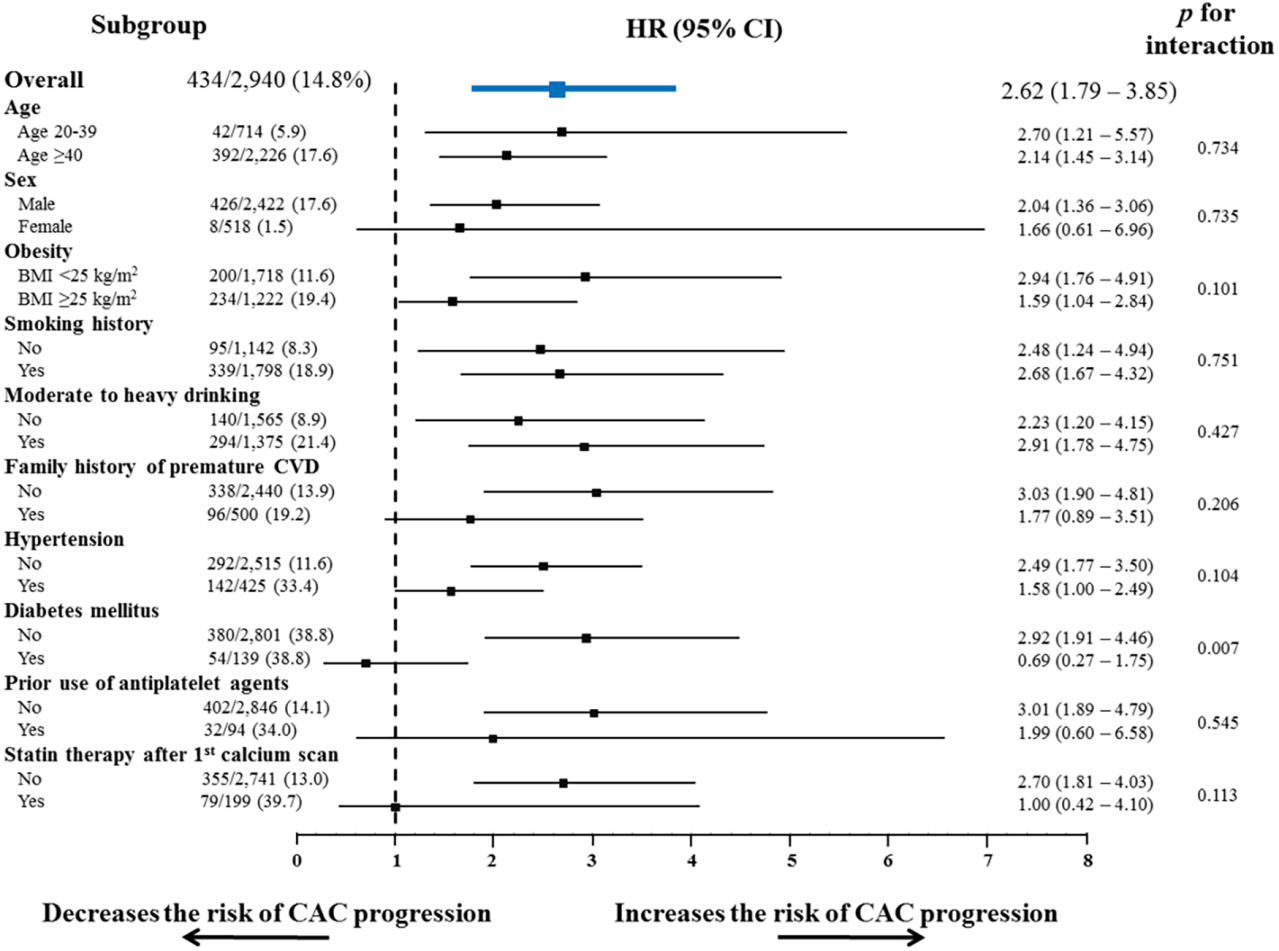
Subgroup analysis of the risk of CAC progression according to conventional risk factors. Non-optimal lipid showed a consistent tendency to increase the risk of CAC progression, in almost all subgroups stratified by conventional cardiovascular risk factors and the related medications. BMI, body mass index; CI, confidence interval; CVD, cardiovascular disease; HR, hazard ratio; other abbreviations as Figure 2.

Subgroup analyses also showed a consistent tendency of non-optimal lipids to increase the risk of CAC progression in almost all the subgroups regarding cardiovascular risk factors and medications (Figure 3), except for groups with diabetes mellitus and those receiving statin therapy after the initial calcium scan.

### 3.3. Risk of CAC progression according to lipid variables

**Figure 4:**
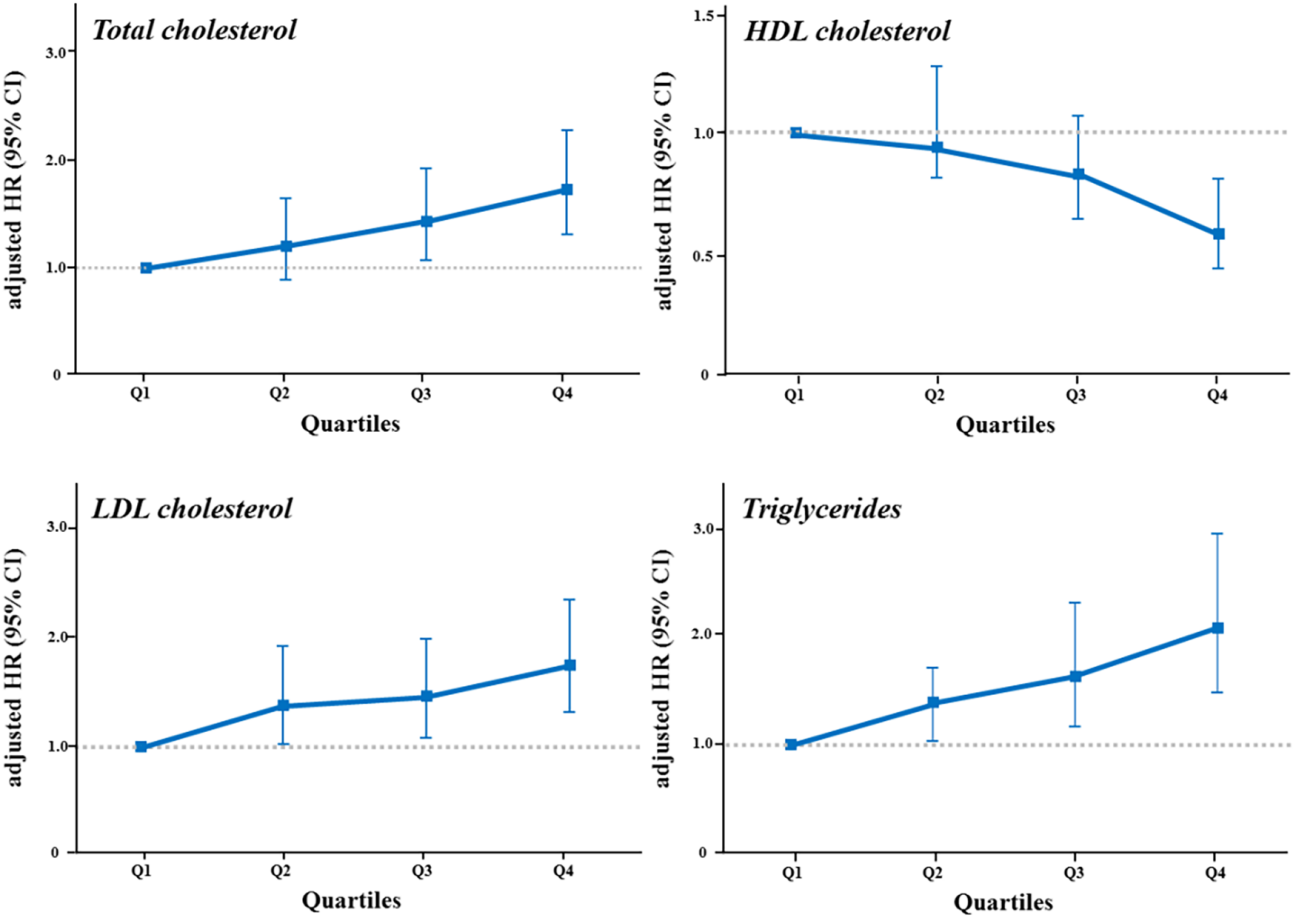
The risk of CAC progression according to quartiles of lipid variables. The risk of CAC progression according to quartiles of each lipid variable was illustrated in a dose-dependent manner by (**A**) total cholesterol, (**B**) HDL cholesterol, (**C**) LDL cholesterol, and (**D**) triglycerides levels. The highest quartile of triglycerides showed the strongest impact on the risk of CAC progression. HDL, high-density lipoprotein; LDL, low-density lipoprotein; other abbreviations as Figures 2, 3.

We additionally analyzed the risk of CAC progression according to the lipid variables. When stratified by quartiles of each lipid variable (Table 3, Figure 4), TC, HDL-C, LDL-C, and triglycerides exhibited a graded association with the incidence and risk of CAC progression, even within the normal ranges. Coronary calcification tended to progress more frequently in participants with higher TC, LDL-C and triglycerides levels, and in those with lower HDL-C levels, after adjusting for conventional risk factors. The risk of CAC progression increased by 72% and 75% in the highest quartiles (Q4) of TC and LDL-C, respectively, compared with the lowest quartile (Q1) as a reference. In contrast, HDL-C Q4 exhibited a 42% lower risk of CAC progression than Q1. Notably, triglycerides Q4 demonstrated the most potent increase in the risk of CAC progression, with a two-fold higher risk than Q1 (adjusted HR 2.08, 95% CI 1.47–2.94, *p* for trend <0.001), even after adjusting for the confounding factors shown the independent associations in the multivariable Cox regression model.

**Table 3:** The incidence and risk of CAC progression according to lipid levels during young adulthood.

Variables (mg/dl)	Event (*n*)	Incidence rate^a^	Adjusted HR (95% CI)^b^
Total cholesterol			
Q1 (≤173)	79	108.8	Reference
Q2 (174–195)	85	115.8	1.20 (0.88–1.64)
Q3 (196–219)	116	155.1	1.43 (1.07–1.92)
Q4 (≥220)	154	210.4	1.72 (1.30–2.27)
***p_trend_***			<0.001
HDL-C			
Q1 (≤42)	133	186.5	Reference
Q2 (43–49)	125	165.6	0.94 (0.81–1.28)
Q3 (50–59)	112	151.9	0.83 (0.64–1.07)
Q4 (≥60)	64	87.1	0.58 (0.43–0.81)
***p_trend_***			0.002
LDL-C			
Q1 (≤98)	66	89.9	Reference
Q2 (99–119)	96	130.8	1.38 (1.01–1.91)
Q3 (120–143)	114	155.3	1.46 (1.08–1.98)
Q4 (≥144)	158	214.1	1.75 (1.31–2.35)
***p_trend_***			0.002
Triglycerides			
Q1 (≤80)	48	65.0	Reference
Q2 (81–119)	84	114.9	1.39 (1.02–1.71)
Q3 (120–177)	129	174.3	1.63 (1.15–2.31)
Q4 (≥178)	173	237.0	2.08 (1.47–2.94)
***p_trend_***			<0.001

^a^ Incidence rate was calculated as 1,000 person-years.

^b^ Model was adjusted for age, sex, obesity, smoking, hypertension, and diabetes mellitus.

Q, quartile; other abbreviations as Tables 1, 2.

### 3.4. CAC progression in the relatively low-risk population

In the relatively low-risk groups of the present cohort, a similar trend of the increased risk of CAC progression was observed in individuals with non-optimal lipid levels. Among participants who did not ensure the optimal lipid levels in their 20 s and 30 s (*n* = 713, 24.3%), non-optimal lipid levels more than doubled the risk of CAC progression (adjusted HR 2.15, 95% CI 1.03–6.22, *p *= 0.041) (Supplementary Table S1). In addition, those with an initial calcium score of zero (*n* = 2,449) had a significantly increased risk of CAC progression (adjusted HR 2.13, 95% CI 1.17–3.87, *p *= 0.014) (Supplementary Table S2). In those without any other cardiovascular risk factors except dyslipidemia (*n* = 676), the risk of CAC progression independently increased by 1.45-fold in the non-optimal lipid group (adjusted HR 1.45, 95% CI 1.03–5.89, *p *= 0.038) (Supplementary Table S3). These results suggest the importance of the non-optimal lipid itself on the progression of CAC, in common.

## 4. Discussion

In this cohort comprising 2,940 young statin-naïve adults aged ≤45 years, the main findings were as follows: (1) participants with an optimal lipid profile accounted for only 16.2% of young adults, indicating that lipid abnormalities develop earlier and more frequently than expected; (2) the presence of non-optimal lipid levels increased the risk of CAC progression by approximately 2.0-fold, with a stepwise risk increase in accordance with lipid abnormality; (3) particularly, this tendency was consistent in the relatively low-risk subgroups, such as those in their 20 s and 30 s, those with an initial CACS of zero, and without other risk factors; and (4) among the lipid variables, triglycerides were an independent and powerful predictor for CAC progression in young adults. Taken together, our findings emphasize the importance of attaining optimal lipid levels from young adulthood in preventing coronary atherosclerosis and also constitute solid evidence supporting current guidelines where the reduction of lifetime exposure to abnormal lipids could promote better cardiovascular health. Consequently, these findings could help establish an appropriate lipid management strategies as primary prevention in young adults.

Uncontrolled cardiovascular risk factors at a young age can lead to considerable differences in the risk of ASCVD later in life (26). Particularly, abnormal lipid levels in young adulthood, as shown in many previous studies (11–13, 27), can increase the risk of mortality and ASCVD. In a prospective study by Klag et al., a strong association between serum cholesterol levels measured in 1,017 young men and subsequent ASCVD later in life was reported during a median follow-up of 30.5 years (27). More recently, a population-based cohort study involving 5,688,055 statin-naïve young adults demonstrated that the risks of death, myocardial infarction, and stroke were proportional to the degree of lipid abnormality over a follow-up of about 7 years (11). All these studies consistently highlight the cumulative effects of exposure to abnormal lipid levels from a young age as a determinant of further ASCVD and death, supporting the recommendations of current guidelines in which timely screening for young adults could be reasonably considered (7, 28). To provide persuasive evidence for these recommendations, researchers have assessed CAC as a surrogate for total coronary atherosclerotic burden in young adults (16, 19, 29). In the CARDIA study, levels of modifiable risk factors, including dyslipidemia in early adults, were significantly informative about the incidence of CAC over two decades later (16, 19). Going one step further, our observations are the first report to verify the strong impact of non-optimal lipid levels on CAC progression assessed by repeated measurements of CACS in young adults. In this study, a significant association was demonstrated between non-optimal lipids and CAC progression despite a rather short follow-up duration (median, 3.3 years), and majority of the final calcium scans were performed in participants before age of 45 years. Therefore, in contrast to the existing data demonstrating the impact of non-optimal lipids at a young age on midlife advanced coronary calcification, we revealed that coronary atherosclerosis could begin and progress earlier in individuals exposed to non-optimal lipids in young adulthood. Additionally, a graded risk increase proportionate to the degree of lipid abnormality was presented, indicating that the risk of CAC progression increased even when lipid levels were within normal range, but not optimal. This was validated in the subgroup analyses on the relatively low-risk groups: very young adults in their 20 s or 30 s, participants with an initial CACS of zero, and those without any other cardiovascular risk factors. Indeed, it can be extended the results from the PESA study (30) in which LDL-C, including at levels currently considered normal, was proportionally associated with the atherosclerotic burden in cardiovascular risk factor-free middle-aged individuals to other lipid variables in young adults. Further, all our findings strongly support the necessity for attainment of optimal lipid levels from young adulthood for cardiovascular health, regardless of an individual’s cardiovascular risk profiles. Particularly, considering that the change in CACS is expected to be greater when the baseline score is higher (31), we adjusted for log formation of (baseline CACS + 1) in the multivariable model and exclusively analyzed the risk of CACS with an initial CACS of zero, demonstrating the importance of optimal lipid levels even in the very low-risk group. We believe that our findings may be of clinical implications for more aggressive management of non-optimal lipid levels as a primary prevention strategy in young adults. Of note, while age showed a significant association with CAC progression in the univariable analysis, it did not demonstrate an independent association in the multivariable analysis in this study. This may be attributed to the characteristics of the current cohort, which consisted of young Korean adults aged ≤45 years, particularly with 20–39 years of age accounting for about 30% of the study participants. Previous studies revealed a linear increase in CAC changes across age, with minimal impact before 50 years old (32–34). In a recent study comprising asymptomatic young adults aged 20–30 years (33), there was no significant difference in mean age between groups with and without CAC, suggesting that age might not have a crucial effect on coronary calcification in this population. Rather, the attenuated effect of age on CAC progression may suggest that emphasis should be placed on prioritizing the management of non-optimal lipids to prevent CAC progression in young adults.

One of our main findings was that triglycerides had a strong effect on CAC progression in young adults. The risk of CAC progression was significantly increased with TC ≥196 mg/dl and LDL-C ≥ 99 mg/dl and decreased with HDL-C ≥ 60 mg/dl, which were currently accepted as the criterion for the optimal lipid levels (23). On the other hand, we demonstrated that CAC was likely to progress when triglycerides exceeded 80 mg/dl, even within the desirable range, and the highest quartile of triglycerides, defined as ≥178 mg/dl, increased the risk of CAC progression by approximately two-fold, compared with the lowest quartile. This is in line with the previous studies, in which triglycerides had a close association with CAC and clinical outcomes in young adults (11, 15, 19), and in which ASCVD risk was increased when triglycerides were >150 mg/dl corresponding to the top quartile of our study (35). In the Muscatine study, triglycerides level measured in young men was significantly related to the incidence of CAC detected ≥15 years later (15). Our group previously demonstrated that triglycerides had an independent and the strongest association with the clinical events, including myocardial infarction and stroke, in a dose-dependent manner among statin-naïve Korean young adults (11). Moreover, it was interesting that a stronger association was noted between triglycerides and CAC progression, rather than LDL-C, which is regarded as the main contributor to coronary atherosclerosis (6, 8). Since evidence is stacking up that the key initiating event in coronary atherosclerosis is the retention of LDL-C in the coronary arterial wall (8), lipid management is primarily targeted at reducing the risk of ASCVD by lowering LDL-C, particularly, in primary prevention (6). The strong predictive value of triglycerides, considered to have low clinical priority so far, could explain the residual risk of ASCVD (36), as well as serve a role as the main screening and potential therapeutic target for young adults (37). As displayed in Table 1, harmful alcohol use, such as heavy episodic drinking or high-risk drinking, is common among Korean young adults (38), and Korean diets have considerably higher carbohydrate levels than Western diets (39). Therefore, the hazard of triglycerides for CAC progression might be more noticeable in this cohort. Additional studies are warranted to generalize our results and to determine whether lipid management, particularly triglycerides, by therapeutic lifestyle modifications including alcohol abstinence and a low-carbohydrate diet, could alleviate the risk of CAC progression in young adults.

### 4.1. Study limitations

This study has some limitations noteworthy to mention. First, the present study derived from the KOICA registry comprises self-referred young adults undergoing repeated calcium scans for routine health check-ups in Korea, and ≥80% of the study participants were men. Hence, the study population may not be fully representative of the general population, and the possibility of selection bias cannot be excluded. However, considering that various subgroup analyses consistently showed that non-optimal lipids were significantly associated with CAC progression, our results were internally validated to be acceptable. It will be required to consider external validation in other populations to ensure generalizability. Second, as the lipid profile was assessed only at baseline, and the subsequent lipid values and clinical practices after the baseline laboratory test were not involved in this study, CAC progression might have been influenced by medical decisions during the follow-up. Considering of paradoxical effect of statin (40), we assessed the proportion of starting statin after the baseline check-up. In our analysis, statin therapy after the initial calcium scan was observed in only 6.8% of study participants, and did not have a significant association with CAC progression. Obviously, it is meaningful in that our results emphasize the importance of cumulative exposure to lipids by presenting the risk of non-optimal lipid levels measured at least once while young adulthood. Third, there might be unidentified confounding factors due to the retrospective nature of the study. Additional prospective studies are required to confirm our results. Fourth, due to the limitation of the study design, detailed data on clinical outcomes were not fully available in the dataset used for this study. Further studies investigating the impact of non-optimal lipids on clinical events, including cardiovascular disease, among young adults will be necessary in the future.

### 4.2. Conclusions

Non-optimal lipids were independently associated with CAC progression, with a gradual risk increase according to lipid abnormality among young adults. Triglycerides emerged as an important screening and potential therapeutic target for preventing CAC progression in young adults. Our findings support that earlier lipid screening and efforts to attain optimal lipid levels from a young age are required to prevent coronary atherosclerosis and to promote better cardiovascular health.

## Data Availability

The raw data supporting the conclusions of this article will be made available by the authors, without undue reservation.
