# Supplementary material for: The effect of non-optimal lipids on the progression of coronary artery calcification in statin-naïve young adults: results from KOICA registry
Source: Front Cardiovasc Med. 2023 Jul 17;10:1173289. doi: 10.3389/fcvm.2023.1173289 (PMC10392939; doi:10.3389/fcvm.2023.1173289)
Supplement: Supplementary file 1 [file Datasheet1.docx]

**Supplementary Table 1. Progression of CAC among the participants in their 20s and 30s (n = 868)**

| **Variables** | **Univariable analysis** | | **Multivariable analysis** | |
| --- | --- | --- | --- | --- |
|  | **Unadjusted HR**  **(95% CI)** | ***p*** | **Adjusted HR**  **(95% CI)** | ***p*** |
| Non-optimal lipid level | 4.86 (1.48 – 19.65) | 0.019 | 2.15 (1.03 – 6.22) | 0.041 |
| Age (per 5 years increment) | 1.38 (0.77 – 2.47) | 0.274 |  |  |
| Male | 4.31 (1.02 – 18.11) | 0.046 | 1.72 (0.84 – 7.56) | 0.475 |
| Obesity | 1.07 (0.54 – 2.12) | 0.849 |  |  |
| Abdominal obesity | 2.42 (1.36 – 7.13) | 0.038 | 1.92 (1.13 – 3.24) | 0.016 |
| Ever smoking | 4.85 (2.02 – 19.17) | 0.008 | 1.04 (1.70 – 9.62) | 0.002 |
| Moderate to heavy drinking | 1.85 (0.80 – 4.25) | 0.150 |  |  |
| Family history of premature CVD | 1.77 (0.80 – 3.94) | 0.162 |  |  |
| Hypertension | 1.51 (0.93 – 2.78) | 0.065 |  |  |
| Diabetes mellitus | 3.57 (0.95 – 8.61) | 0.075 |  |  |
| Statin after 1^st^ calcium scan | 1.43 (0.32 – 6.37) | 0.639 |  |  |
| hs-C reactive protein ≥2.0 mg/L | 1.93 (0.40 – 9.21) | 0.410 |  |  |
| Log (baseline CACS + 1) | 2.71 (2.04 – 3.60) | <0.001 | 2.43 (1.81 – 3.26) | <0.001 |

Multivariable analysis was adjusted for significant risk factors in the univariable analysis. CAC, coronary artery calcification; CCTA, coronary computed tomography angiography; CI, confidence interval; CVD, cardiovascular disease; HR, hazard ratio; hs, high-sensitivity.

**Supplementary Table 2. Progression of CAC among the participants with an initial calcium score of zero (n = 2,449)**

| **Variables** | **Univariable analysis** | | **Multivariable analysis** | |
| --- | --- | --- | --- | --- |
|  | **Unadjusted HR**  **(95% CI)** | ***p*** | **Adjusted HR**  **(95% CI)** | ***p*** |
| Non-optimal lipid level | 2.57 (1.43 – 4.64) | 0.002 | 2.13 (1.17 – 3.87) | 0.014 |
| Age (per 5 years increment) | 1.26 (0.99 – 1.61) | 0.060 |  |  |
| Male | 5.17 (1.91 – 14.01) | 0.001 | 2.82 (1.00 – 7.94) | 0.049 |
| Obesity | 1.34 (0.98 – 1.84) | 0.068 |  |  |
| Abdominal obesity | 1.74 (1.22 – 2.43) | 0.002 | 1.39 (0.98 – 1.99) | 0.067 |
| Ever smoking | 1.65 (1.12 – 2.43) | 0.011 | 1.34 (0.89 – 2.01) | 0.156 |
| Moderate to heavy drinking | 1.21 (0.85 – 1.73) | 0.298 |  |  |
| Family history of premature CVD | 1.27 (0.88 – 1.85) | 0.205 |  |  |
| Hypertension | 2.23 (1.57 – 3.17) | <0.001 | 1.96 (1.36 – 2.80) | <0.001 |
| Diabetes mellitus | 1.86 (1.10 – 3.13) | 0.020 | 1.43 (0.84 – 2.43) | 0.183 |
| Statin after 1^st^ calcium scan | 1.18 (0.74 – 1.86) | 0.490 |  |  |
| Systolic BP ≥140 mmHg | 1.98 (1.23 – 3.17) | 0.005 |  |  |
| Glucose ≥100 mg/dL | 1.76 (1.25 – 2.48) | 0.001 |  |  |
| hs-C reactive protein ≥2.0 mg/L | 1.73 (0.90 – 3.33) | 0.101 |  |  |

Multivariable analysis was adjusted for significant risk factors in the univariable analysis. Abbreviations as Supplementary Table 1.

**Supplementary Table 3. Progression of CAC among the participants without other cardiovascular risk factors except non-optimal lipid levels (n = 592)**

| **Variables** | **Univariable analysis** | | **Multivariable analysis** | |
| --- | --- | --- | --- | --- |
|  | **Unadjusted HR**  **(95% CI)** | ***p*** | **Adjusted HR**  **(95% CI)** | ***p*** |
| Non-optimal lipid level | 2.10 (1.23 – 4.66) | 0.009 | 1.45 (1.03 – 5.89) | 0.038 |
| Age (per 5 years increment) | 1.44 (0.93 – 3.26) | 0.080 |  |  |
| Male | 8.77 (1.11 – 22.03) | 0.037 | 1.72 (0.64 – 4.65) | 0.285 |
| BMI | 1.02 (0.87 – 1.19) | 0.814 |  |  |
| Systolic BP ≥120 mmHg | 1.93 (1.37 – 6.45) | 0.001 | 1.28 (1.03 – 1.60) | 0.025 |
| Glucose ≥100 mg/dL | 1.37 (0.94 – 2.83) | 0.084 |  |  |
| hs-C reactive protein ≥2.0 mg/L | 2.27 (0.28 – 8.29) | 0.439 |  |  |
| Log (baseline CACS + 1) | 2.66 (2.36 – 2.99) | <0.001 | 2.56 (2.26 – 2.90) | <0.001 |

Multivariable analysis was adjusted for significant risk factors in the univariable analysis. BMI, body mass index; other abbreviations as Supplementary Table 1.

**SUPPLEMENTAL FIGURES**

**Supplementary Figure 1. The distribution of CACS at baseline and follow-up calcium scans**

**
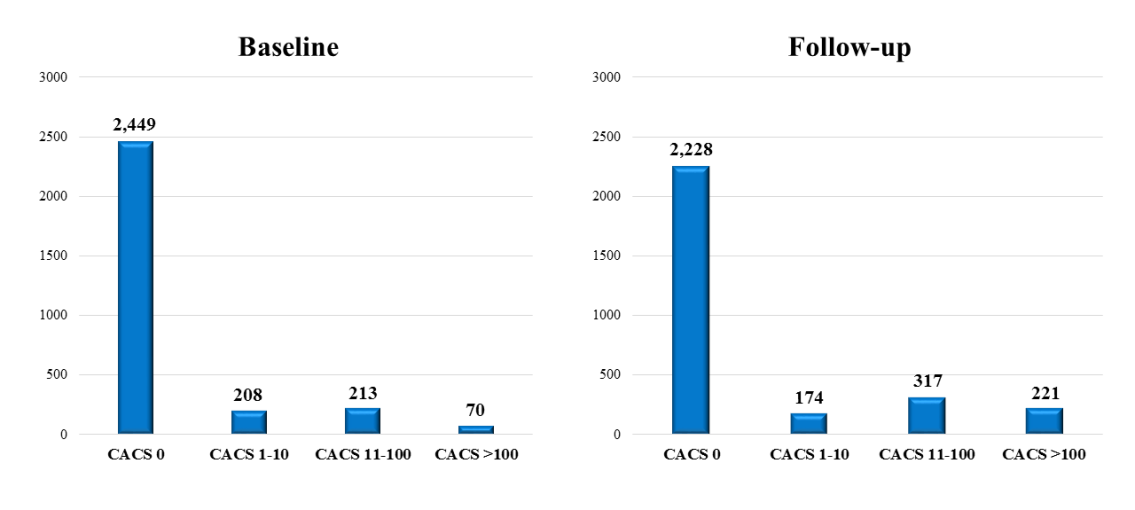
**

CACS, coronary artery calcium scores.
